# An Ecological Study of Tuberculosis Incidence in China, From 2002 to 2018

**DOI:** 10.3389/fpubh.2021.766362

**Published:** 2022-01-18

**Authors:** Qianyun Zhang, Wanmei Song, Siqi Liu, Qiqi An, Ningning Tao, Xuehan Zhu, Dongmei Yang, Daoxia Wan, Yifan Li, Huaichen Li

**Affiliations:** ^1^Department of Respiratory Medicine, Cheeloo College of Medicine, Shandong Provincial Hospital, Shandong University, Jinan, China; ^2^Department of Respiratory Medicine, Shandong Provincial Hospital Affiliated to Shandong First Medical University, Jinan, China; ^3^College of Statistics, Shandong University of Finance and Economics, Jinan, China; ^4^Shandong University of Traditional Chinese Medicine, Jinan, China

**Keywords:** tuberculosis, incidence, factors, ecological study, China

## Abstract

**Introduction:**

Tuberculosis is one of the main infectious diseases threatening global health, which is also the main cause of death from a single source of infection (above HIV/AIDS). China is a country with a high burden of tuberculosis in the world, ranking only behind India and Indonesia. However, there are few ecological studies on the burden of tuberculosis in China. This study aims to provide more research basis for the government to formulate tuberculosis policies by exploring the ecological factors associated with the incidence of tuberculosis, so as to achieve the goal of eliminating tuberculosis by 2030.

**Methods:**

We collected data on the incidence of tuberculosis and ecological factors of 31 provinces, autonomous regions, and municipalities in Mainland China (excluding Taiwan, Hong Kong, and Macau) from 2002 to 2018. We constructed a framework of ecological factors affecting the incidence, which consists of 5 secondary indicators and 35 tertiary indicators. And we construct a dynamic panel data model based on the Lasso Regression to select variables to test the effect of each ecological factor on the incidence.

**Results:**

Among the 35 tertiary indicators, economy 3,4,6,7, environment 1, recourses 1,3, demography 3, and lifecare 2,4,8,9,13 passed the significance test at the 1% level, economy 1,2,5, environment 2,9, lifecare 6,12 passed the significance test at the 5% level, lifecare 10 passed the significance test at the 10% level. Only economy 5 and economy 6 have a positive impact on the incidence, other statistically significant ecological indicators are negatively correlated with the incidence.

**Conclusions:**

Our study indicated that many ecological factors, including residents' income, unemployment rate, educational level, medical resources, population density, sunshine duration and dietary structure, are closely related to the incidence of tuberculosis. These findings contribute the government to taking targeted measures for tuberculosis prevention and control, including improving the level of economic development, increasing employment, expanding the scale of enrollment in colleges and universities, and ensuring that the prices of sources of animal protein are reasonable to meet the residents' intake of protein.

## Introduction

Tuberculosis (TB) is one of the top 10 causes of death worldwide and the leading cause of death from a single infectious agent (ranking above HIV/AIDS)—infecting around 10 million people and causing 1.4 million deaths in 2019 (1). Although the Millennium Development Goal of halving TB prevalence and mortality by 2015, compared to 1990 levels, has been achieved around the world (2). However, to achieve one of the Sustainable Development Goals (SDGs) issued by the United Nations (UN)—to eliminate tuberculosis by 2030, we still have a long and difficult road to go (3). Especially since 2019, COVID-19 has been raging around the world, which is bound to cause health system infrastructure pivot toward COVID-19 and away from competing illnesses, including tuberculosis (4). According to the report of Chen and Zhang, the COVID-19 pandemic is predicted to increase tuberculosis deaths globally by 20% over the next 5 years (5).

China is one of the 30 countries with a high burden of tuberculosis in the world, with tuberculosis cases accounting for 8.4% of the world, ranking only behind India (26%) and Indonesia (8.5%) (1). In the 1990s, the government implemented two major tuberculosis control projects, including the implementation of the directly observed treatment of short course strategy (DOTS) in 13 provinces from 1992 to 2001 and the use of limited funds from the Ministry of Health to fund treatment of an additional 10–15% of the population. These measures have significantly reduced the prevalence and mortality of tuberculosis in China (6, 7). However, in the current international context, the prevention and control of tuberculosis still face huge challenges. In order to achieve the SDG target to end the tuberculosis epidemic by 2030, robust assessments of tuberculosis incidence are essential for making polices.

For tuberculosis, a preventable, detectable and treatable infectious disease, the focus is on prevention, especially when drug-resistant tuberculosis becomes a major public health threat. If we can find out factors closely related to the incidence of tuberculosis, the prevention and control of tuberculosis will be of great help. There are many foreign studies on the association between the burden of tuberculosis and influencing factors. Their researches have found that increasing social protection and public health expenditure is closely related to reducing the incidence, mortality (8–10), but whether it is applicable to China is open to question. The purpose of this study is to identify ecological factors associated with the incidence of tuberculosis through correlation analysis, so as to fulfill the task of preventing and treating TB better.

## Methods

In this study, we selected some ecological factors affecting the incidence of TB and construct a dynamic panel data model based on the Least Absolute Shrinkage and Selection Operator (Lasso Regression) to select variables to test the effect of each ecological factor on the incidence of TB.

### Data Sources

We selected some indicators based on previous international studies on the relationship between TB incidence and economic development, social protection and other factors. These factors is composed of 5 secondary indicators, including socio-economic development, health resources, demography, living environment, and residents' health care and 35 tertiary indicators. According to the data in China Statistical Yearbook, we have retained the indicators that can be found in the statistical yearbook and excluded indicators that are not recorded in the statistical yearbook. Table 1 shows the full list of ecological factors. In this study, we collected sample data of 31 provinces, autonomous regions, and municipalities (hereafter, provinces) in mainland China (Taiwan, Hong Kong, and Macao are not included) from 2002 to 2018. The data on the incidence of TB and ecological factors are all from the “China Statistical Yearbook” (11). In addition, we used the interpolation method to replace individual missing values and used software STATA 16.0 for statistics.

**Table 1:** The framework of ecological factors influencing the incidence of pulmonary tuberculosis.

**First level indicator**	**Secondary indicators**	**Third level indicators**	**Variable**
	Socio-economic development	Urban per capita disposable income /yuan	Economy 1
		Rural per capita disposable income/yuan	Economy 2
		GDP per capita/yuan	Economy 3
		Proportion of urban population/%	Economy 4
		unemployment rate/%	Economy 5
		Proportion of the illiterate population in the population aged 15 and above/%	Economy 6
		Average number of students enrolled in higher education per 100,000 population	Economy 7
	Health resources	Number of beds in medical and health institutions per 1,000 population in the city	Resources 1
		Number of beds in medical and health institutions per 1,000 population in rural areas	Resources 2
		Number of practicing physicians per 1,000 population	Resources 3
	Demography	Population at the end of the year/10,000 people	Demography 1
		Gender ratio (female=100)	Demography 2
		Urban population density (person/km^2^)	Demography 3
Factors affecting the TB incidence	Living environment	Public transportation vehicles per 10,000 people	Environment 1
		Total wastewater discharge/10,000 tons	Environment 2
		Waste gas sulfur dioxide emissions/10,000 tons	Environment 3
		Exhaust gas smoke (powder) dust emission/10,000 tons	Environment 4
		Annual average concentration of PM10 (ug/m^3^)	Environment 5
		Annual average temperature/°C	Environment 6
		Annual average relative humidity/%	Environment 7
		Annual precipitation/mm	Environment 8
		Annual sunshine hours	Environment 9
	Lifecare	Urban per capita health care expenditure/yuan	Lifecare 1
		Rural per capita health care expenditure/yuan	Lifecare 2
		Urban health care expenditures accounted for consumption expenditures/%	Lifecare 3
		Rural health care expenditures accounted for consumption expenditures/%	Lifecare 4
		Per capita vegetable consumption of rural households/Kg	Lifecare 5
		Per capita consumption of meat (pig, beef, mutton) in rural households/Kg	Lifecare 6
		Per capita consumption of poultry in rural households/Kg	Lifecare 7
		Per capita consumption of aquatic products in rural households/Kg	Lifecare 8
		Per capita egg consumption of rural households/Kg	Lifecare 9
		Average annual vegetable consumption expenditure per capita of urban households/yuan	Lifecare 10
		Average annual consumption expenditure of meat, poultry and products per person in urban households/yuan	Lifecare 11
		Average annual consumption of aquatic products per person in urban households/yuan	Lifecare 12
		Average annual egg consumption expenditure per person in urban households/yuan	Lifecare 13

*TB, tuberculosis; GDP, gross domestic product*.

### Variable Selection

We separately examine the impact of ecological factors on the incidence in five aspects: social and economic development, health resources, demography, living environment, and residents' health care. Each aspect contains multiple three-level indicator variables, and there is multiple collinearity among the variables. If we directly incorporate these indicators into the model, it will lead to estimation bias. Therefore, we use the Lasso Regression to analyze the variables and make a selection. The basic theory of Lasso regression is to make the partial regression coefficients estimated at 0 by the maximum likelihood target function under the constraint that the sum of the absolute values of the regression coefficient is less than one parameter lambda, so as to delete the unimportant variables and improve the explanatory power of the model. We determined the value of lambda by cross-validation, adaptive and plugin, and minimized the mean squared error (MSE). The results of the selection of five ecological aspects are shown in Table 2. The variables, including environment 8, lifecare 1, lifecare 7 and resources 2, are rejected by Lasso Regression.

**Table 2:** Variable results filtered by Lasso method.

**Secondary indicators**	**SELECTION**	**LOPT**	**MSE**	**NO**.	**PE**
Socio-economic development	Cross Validation	0.0025	0.6069	7	0.8179
Health resources	Adaptive	0.0146	0.725	2	0.9358
Demography	Adaptive	0.0016	0.8049	3	1.0243
Living environment	Cross Validation	0.0123	0.7005	8	0.9875
Lifecare	Adaptive	0.009	0.5029	11	0.577

*LOPT means choosing the lambda that minimizes MSE; NO, indicates the number of coefficient estimates that are not 0; PE represents the mean prediction error of CV*.

### Model Construction and Estimation

We took the incidence of TB as the dependent variable and each ecological factor as the independent variable, and constructed the following dynamic panel data model:


$$\begin{array}{l} {\text{y}}_{\text{it}}=\alpha_{1}+\rho_{1}{\text{y}}_{\text{it}-1}+\beta_{\text{1k}}{\text{economy}}_{\text{kit}}+{\text{u}}_{\text{i}}+\varepsilon_{\text{it}}\\ {\text{y}}_{\text{it}}=\alpha_{2}+\rho_{2}{\text{y}}_{\text{it}-1}+\beta_{\text{2k}}{\text{resources}}_{\text{kit}}+{\text{u}}_{\text{i}}+\varepsilon_{\text{it}}\\ {\text{y}}_{\text{it}}=\alpha_{3}+\rho_{3}{\text{y}}_{\text{it}-1}+\beta_{\text{3k}}{\text{demography}}_{\text{kit}}+{\text{u}}_{\text{i}}+\varepsilon_{\text{it}}\\ {\text{y}}_{\text{it}}=\alpha_{4}+\rho_{4}{\text{y}}_{\text{it}-1}+\beta_{\text{4k}}{\text{environment}}_{\text{kit}}+{\text{u}}_{\text{i}}+\varepsilon_{\text{it}}\\ {\text{y}}_{\text{it}}=\alpha_{5}+\rho_{5}{\text{y}}_{\text{it}-1}+\beta_{\text{5k}}{\text{lifecare}}_{\text{kit}}+{\text{u}}_{\text{i}}+\varepsilon_{\text{it}} \end{array}$$

In the formula, y_it_ is the incidence of TB in area i in year t; y_it−1_ is a lagging variable; economy_kit_, resources_kit_, demography_kit_, environment_kit_, and lifecare_kit_ are the socio-economic development variables, health resource variables, and demographic variables, living environment variables and residents' health care variables. k represents the k-th three-level indicator variable; μ_i_ is an unobservable individual effect that does not change with time; ε_it_ is a random disturbance item; i is the province in mainland China; t is time, and the unit is year.

Based on the panel data of inter-provincial tuberculosis incidence and ecological indicators from 2002 to 2018, we constructed a dynamic panel data system and used System Generalized Method of Moments (System GMM) to estimate. The System GMM model considers the endogenous problem caused by the lag term y_it−1_. To a certain extent, this model can overcome the bias compared to static models because static models ignore the endogenous problems. The *P*-value was set as 5% in the study. The consistency of System GMM estimate depends on two conditions. The first condition is that there is no self-correlation for perturbation terms, which is tested with AR (1) and AR (2). The second condition is that the variables are valid, which is tested with the Sargan-Hansen statistics. First, System GMM requires that the first-order differential of the perturbation item is self-correlated, which requires that the *P*-value of AR(1) is <0.1, but it does not allow self-correlation in the second-order differential of the perturbation term at the same time, which need the *P*-value of AR(2) be >0.1. The results of the model estimates are shown in Tables 3–**6**. As seen from the tables, the AR(1) test passed the 5% significance test and the AR(2) test failed the 10% significance test (the *P*-value of AR(1) test <0.1, and the *P*-value of AR(2) test >0.1), indicating that there were no sequence-related problems of the random interference items in the model. Second, the Sargan-Hansen test is an over-recognition test. The assumption is that all tool variables are exogenous, which means there is no transition recognition. The results in Tables 3–**6** show that the *P*-value of the Sargan-Hansen test is greater than the 5% significance level, so that the original hypothesis cannot be rejected. This indicates that there is no transition recognition phenomenon in the model, and the variables selected by the model are reasonable. Both conditions of the System GMM are satisfied, indicating that the System GMM is a consistent estimate and has practical reference significance.

**Table 3:** Estimation results of the impact of socio-economic development variables on the incidence of tuberculosis.

**Model**	**(1)**	**(2)**	**(3)**	**(4)**	**(5)**	**(6)**	**(7)**
**Dependent variable**	**The incidence of tuberculosis: y**						
**Independent variables**	**Economy 1**	**Economy 2**	**Economy 3**	**Economy 4**	**Economy 5**	**Economy 6**	**Economy 7**
L.y	0.4666^***^	0.4395^***^	0.4494^***^	0.4211^***^	0.5308^***^	0.5291^***^	0.5052^***^
	(0.0643)	(0.0650)	(0.0618)	(0.0650)	(0.0823)	(0.0738)	(0.0587)
Economy	−0.1916^**^	−0.2197^**^	−0.2652^***^	−0.4443^***^	0.1701^**^	0.0761^***^	−0.2485^***^
	(0.0828)	(0.0941)	(0.0753)	(0.1078)	(0.0713)	(0.0291)	(0.0787)
Constant	0.0434	0.0580	0.0611	0.0411	0.0413	0.0464	0.0646
	(0.0932)	(0.0853)	(0.0835)	(0.0660)	(0.0828)	(0.0750)	(0.0718)
Number	496	496	496	496	490	496	496
AR (1) test	0.0148	0.0171	0.016	0.0167	0.0168	0.0161	0.0161
AR (2) test	0.2107	0.2321	0.2421	0.2386	0.2394	0.2602	0.2236
Sargan-Hansen test	1.0000	1.0000	1.0000	1.0000	1.0000	1.0000	1.0000

*L.y, the lag 1 period of y*.

*L.y, economy, and constant correspond to two rows of values: the first row is the estimated value of the coefficient, and the second row is the standard error (SE) in parentheses*.

*, **, *** *indicates that the data is significant at the level of 10, 5, and 1% respectively*.

*+ and – represent positive correlation and negative correlation, respectively*.

*Number represents all the sample values entered into the model*.

## Results

We studied the relationship between 35 ecological variables and the incidence of TB in China from 2002 to 2018. And four variables (environment 8, lifecare 1, lifecare 7 and resources 2) are eliminated by the Lasso Regression method.

The results in Table 3 show that among the socio-economic development variables, economy 3,4,6,7 passed the significance test at the 1% level and economy 1,2,5 passed the significance test at the 5% level. Economy 1,2,3,4,7 all have a significant negative impact on the incidence of TB. In other words, with the increase of urban and rural per capita disposable income, GDP per capita, the proportion of urban population, and the number of people with higher education, the incidence of TB has shown a significant downward trend. Economy 5 and 6 have a significant positive impact on the incidence of TB.

The results in Table 4 show that among the living environment variables, environment 1 passed the significance test at the 1% level, environment 2 and 9 passed the significance test at the 5% level. And these three variables are negatively correlated with the incidence of TB. With the increase in public transport vehicles per 10,000 people, total waste water discharge, and annual sunshine duration, the incidence of TB has shown a significant downward trend. However, the impact of other environmental variables on the incidence is not statistically significant, so we will not discuss it in this study.

**Table 4:** Estimation results of the impact of living environment variables on the incidence of tuberculosis.

**Model**	**(1)**	**(2)**	**(3)**	**(4)**	**(5)**	**(6)**	**(7)**	**(8)**
**Dependent variable**	**The incidence of tuberculosis: y**							
**Independent variables**	**Envir_1**	**Envir_2**	**Envir_3**	**Envir_4**	**Envir_5**	**Envir_t6**	**Envir_7**	**Envir_9**
L.y	0.5411^***^	0.4881^***^	0.5244^***^	0.5467^***^	0.5272^***^	0.5722^***^	0.5470^***^	0.5483^***^
	(0.0493)	(0.0715)	(0.0798)	(0.0863)	(0.0912)	(0.0734)	(0.0808)	(0.0820)
Environment	−0.3333^***^	−0.1980^**^	0.0823	−0.0077	−0.0889	0.0790	−0.0064	−0.1499^**^
	(0.0859)	(0.0879)	(0.0747)	(0.0336)	(0.0634)	(0.0862)	(0.0748)	(0.0659)
Constant	0.0572	0.0487	0.0357	0.0408	0.0336	0.0434	0.0457	0.0402
	(0.0816)	(0.0844)	(0.0794)	(0.0758)	(0.0718)	(0.0731)	(0.0744)	(0.0864)
Number	496	496	496	496	496	496	496	496
AR (1) test	0.0115	0.0158	0.0154	0.0156	0.0169	0.0147	0.0158	0.0148
AR (2) test	0.3365	0.2073	0.1924	0.1896	0.1949	0.1871	0.189	0.1956
Sargan-Hansen test	1.000	1.000	1.000	1.000	1.000	1.000	1.000	1.000

**, *** *indicates that the data is significant at the level of 5% and 1% respectively*.

The results in Table 5 show that among the health resources and demographic variables, recourses 1,3 and demography 3 passed the significance test at the 1% level, and these three variables are all negatively correlated with the incidence of TB. That is to say, with the increase in the number of beds in medical and health institutions, the number of practicing physicians and urban population density, the incidence of TB has decreased significantly. But there was no significant statistical significance between the remaining demographic variables and the incidence.

**Table 5:** Estimated results of the impact of health resources and demographic variables on the incidence of tuberculosis.

**Model**	**(1)**	**(2)**	**Model**	**(1)**	**(2)**	**(3)**
**Dependent variable**	**The incidence of TB: y**		**Dependent variable**	**The incidence of TB: y**		
**Independent variables**	**Resources 1**	**Resources 3**	**Independent variables**	**Demography 1**	**Demography 2**	**Demography 3**
L.y	0.4916^***^	0.4041^***^	L.y	0.4907^***^	0.5211^***^	0.6099^***^
	(0.0432)	(0.0439)		(0.0840)	(0.0616)	(0.0895)
Resources	−0.2574^***^	−0.3503^***^	Demography	−0.1433	−0.0403	−0.2641^***^
	(0.0340)	(0.0571)		(0.1447)	(0.0541)	(0.0746)
Constant	0.0534	0.0277	Constant	0.0389	0.0427	0.0492
	(0.0907)	(0.0868)		(0.0812)	(0.0777)	(0.0799)
Number	496	496	Number	496	496	496
AR (1) test	0.0114	0.0133	AR (1) test	0.0175	0.0152	0.0175
AR (2) test	0.1051	0.216	AR (2) test	0.2083	0.1868	0.0943
Sargan-Hansen test	1.0000	1.0000	Sargan-Hansen test	1.0000	1.0000	1.0000

*** *indicates that the data is significant at the level of 1%*.

The results in Table 6 show that among the lifecare variables, lifecare 2,4,8,9,13 passed the significance test at the 1% level, lifecare 6,12 passed the significance test at the 5% level and lifecare 10 passed the significance test at the 10% level. And the above variables are significantly negatively correlated with the incidence of TB. These factors include rural per capita health care expenditure and its proportion in consumer expenditure, the consumption of meat, aquatic products and egg in rural areas, urban household aquatic products and egg consumption expenditure, all of which can significantly reduce the incidence of TB. The vegetable consumption in urban households also has a significant effect on reducing the incidence of TB. But the statistical significance of other lifecare variables on the incidence is not obvious.

**Table 6:** Estimated results of the impact of lifecare variables on the incidence of tuberculosis.

**Model**	**(1)**	**(2)**	**(3)**	**(4)**	**(5)**	**(6)**
**Dependent variable**	**The incidence of TB: y**					
**Independent variables**	**Lifecare 2**	**Lifecare 3**	**Lifecare 4**	**Lifecare 5**	**Lifecare 6**	**Lifecare 8**
L.y	0.4261^***^	0.5251^***^	0.4649^***^	0.5102^***^	0.5165^***^	0.4799^***^
	(0.0545)	(0.0626)	(0.0655)	(0.0808)	(0.0473)	(0.0513)
Lifecare	−0.2582^***^	−0.1445	−0.4397^***^	0.1812	−0.2274^**^	−0.3182^***^
	(0.0339)	(0.1737)	(0.0630)	(0.1302)	(0.0982)	(0.1017)
Constant	0.0585	0.0366	0.0596	0.0480	0.0472	0.0349
	(0.0865)	(0.0774)	(0.0968)	(0.0852)	(0.0960)	(0.0860)
Number	496	496	496	496	496	496
AR (1) test	0.0163	0.0139	0.0164	0.0159	0.0153	0.014
AR (2) test	0.2384	0.2716	0.2315	0.1897	0.133	0.1887
Sargan-Hansen test	1.0000	1.0000	1.0000	1.0000	1.0000	1.0000
**Model**		**(7)**	**(8)**	**(9)**	**(10)**	**(11)**
**Dependent variable**		**The incidence of TB: y**				
**Independent variables**		**lifecare 9**	**lifecare 10**	**lifecare 11**	**lifecare 12**	**lifecare 13**
L.y		0.3751^***^	0.5060^***^	0.5235^***^	0.4443^***^	0.4747^***^
		(0.0807)	(0.0741)	(0.0767)	(0.0640)	(0.0612)
Lifecare		−0.5408^***^	−0.0921^*^	−0.0520	−0.1727^**^	−0.1883^***^
		(0.0903)	(0.0522)	(0.0696)	(0.0706)	(0.0459)
Constant		0.0616	0.0412	0.0436	0.0402	0.0591
		(0.0734)	(0.0857)	(0.0833)	(0.0913)	(0.0765)
Number		496	496	496	496	496
AR (1) test		0.0208	0.0157	0.0156	0.0164	0.0147
AR (2) test		0.3296	0.2003	0.2121	0.2227	0.2583
Sargan-Hansen test		1.0000	1.0000	1.0000	1.0000	1.0000

*,**, *** *indicate that the data is significant at the level of 10%, 5% and 1% respectively*.

## Discussion

As far as we know, ecological researches on the burden of tuberculosis have been carried out globally, such as the Americas, Europe and Africa (8–10, 12), but such research is limited in China. Only by finding factors closely related to the incidence of tuberculosis can we do a better job in tuberculosis prevention and control. In this study, we expanded the scope of the data to the national level and found that many of these factors can obviously affect the incidence of TB, including economy 1 to economy 7, environment 1,2,9, recourses 1,3, demography 3 and lifecare 2,4,6,8,9,10,12,13.

The factors, from economy 1 to economy 7, are closely related to the level of economic development. As a result of economic development, urban and rural per capita disposable income, per capita GDP, the proportion of urban population, the number of people with higher education have increased, and the unemployment rate and illiteracy rate have decreased. In this way, people's living standards and health care awareness will be improved, which can reduce the risk of TB to a certain extent. These results corroborate the findings of a previous global analysis that social protection spending is strongly associated with lower tuberculosis case notification, incidence, and mortality rates (13). And our results also confirm the report that economic development and increased access to health care both reduce TB incidence (14). Therefore, the government should vigorously develop the economy, expand the scale of college enrollment and increase employment opportunities. However, economic development may take a long time, which is the drawback of our research. In the future research, we should choose the convertible indicators for analysis, so that the research will have greater practicality.

Environment 1 and 2, refer to the public transport vehicles per 10,000 people and total waste water discharge, which can significantly reduce the incidence of TB. Although these two are classified as environmental factors, they are also related to economic development. In economically developed areas, the government will invest more in public transportation. Therefore, the more public transportation vehicles a unit of population has, the lower the population density in the vehicles. At the same time, our research also confirmed that demography 3, the urban population density, is significantly negatively correlated with the incidence. These conclusions confirm previous researches that the increased population density increases the burden of TB (15, 16). This conclusion is of great significance for the control of pulmonary tuberculosis, an infectious disease that is mainly transmitted by respiratory droplets. The total waste water discharge, includes domestic wastewater and industrial wastewater. To a certain extent, the amount of waste water discharge reflects superior living conditions and developed industries, and the level of industrial development is also an important indicator for measuring regional economic development. Therefore, the government should close down high-polluting enterprises and develop green and energy-saving economic enterprises. Environment 9 is annual sunshine duration, which is negatively correlated with the incidence of TB. There are many studies proving that sunshine and vitamin D levels are associated with the incidence of tuberculosis (17–20). Sunshine exposure is necessary for the production of vitamin D by the body and vitamin D plays a role in the host response to Mycobacterium tuberculosis. We speculate that the sunshine could also affect the time-spent-indoors (aerosol exposure) as well as the environmental survival of Mycobacterium tuberculosis.

Recourses 1 and 3, meaning the number of beds in medical institutions and the number of practicing physicians, have a significant negative correlation with the incidence. This conclusion is consistent with the result of a previous global analysis that the level of health expenditure is negatively correlated with the incidence of tuberculosis (13). However, our research is more detailed, and further explored the impact of the number of beds and the number of physicians on the incidence of TB. Therefore, relevant government departments should increase investment in medical and health care, including increasing hospital beds and expanding the scale of medical student enrollment, so as to provide hospitals with more physician resources.

Lifecare 2,4,6,8,9,10,12 and 13 are all negatively correlated with the incidence of TB. The results show that increasing medical and health care expenditures have far more impact on reducing the incidence of TB among rural residents than urban residents. We believe that because rural residents have relatively poor health care awareness and low income, they will not seek medical attention unless the symptoms of the disease are very obvious, which will inevitably cause delays in the diagnosis and treatment of tuberculosis (21, 22). However, delays in diagnosis and treatment will increase the chance of tuberculosis patients infecting susceptible people around them, which eventually leads to an increase in incidence. This finding helps the government shift the focus of tuberculosis prevention and control to rural areas and carry out relevant work in a targeted manner. In addition, dietary structure also has a certain impact on the incidence of TB. Both rural and urban residents should ensure the intake of aquatic products and eggs, while rural residents should also have sufficient meat intake. We consider the influence of dietary structure on incidence mainly because meat, aquatic products and eggs are rich in protein and vitamin D (18). The dietary sources of vitamin D are mainly fish, eggs, and butter. As mentioned earlier, vitamin D plays a role in the host response to Mycobacterium tuberculosis. So the health department can develop appropriate brochures about the dietary structure and provide guidance on nutritional matching.

Previous studies only explored the relationship between medical expenditures and the burden of tuberculosis, while our research is more detailed, specifically the impact of the number of beds and the number of doctors on the incidence of TB. But there are still some limitations in our research. First of all, the burden of tuberculosis is not only reflected in the incidence, but also includes prevalence and mortality. However, due to the limited data we can collect, we can only conduct ecological studies on the incidence of TB. Moreover, some of the ecological indicators designed in this study are indeed difficult to change, such as population, gender ratio, especially temperature, humidity, sunshine duration, precipitation and other indicators in environmental factors. In the future research, we should choose the convertible indicators for analysis, so that the research will have greater practicality. In addition, tuberculosis is preventable and controllable no matter at the group or individual level, but our research only considers the group. If we can analyze the correlation between individual behavior and the burden of TB at the individual level in the future, it will help all people participate in the prevention and treatment of tuberculosis, not just the government.

## Conclusion

Although the current pandemic of COVID-19 has reversed the progress of tuberculosis, the experience of COVID-19 also shows that political will and investments can prompt extraordinary public health actions and scientific advances against infectious diseases. In summary, we have drawn the following conclusions that factors such as residents' income, unemployment rate, educational level, medical resources, population density, sunshine duration and dietary structure are closely related to the incidence of tuberculosis. The results of this study will help the government to take targeted measures for tuberculosis prevention and control, including improving the level of economic development, increasing employment, expanding the scale of enrollment in colleges and universities, and ensuring that the prices of sources of animal protein are reasonable to meet the residents' intake of protein, thereby can we achieve the goal of eliminating tuberculosis by 2030 faster and better.

## Data Availability Statement

The original contributions presented in the study are included in the article/Supplementary Material, further inquiries can be directed to the corresponding author/s.

## Author Contributions

HL and QZ conceived and designed the study. HL, DY, WS, and SL directed its implementation including the data analysis and writing of the paper. QZ, DW, and QA analyzed the data. XZ, NT, and YL contributed materials/analytic tools. QZ, YL, and HL wrote and revised the manuscript. All authors reviewed and approved the manuscript.

## Funding

This study is a project supported and developed by the government. It was financed by Department of Science & Technology of Shandong Province (CN) (No. 2007GG30002033; No.2017GSF218052) and Jinan Science and Technology Bureau (CN) (No. 201704100). Department of Science & Technology of Shandong Province provided financial support during our data collection. Jinan Science and Technology Bureau financed the purchase of software for the study. The funders had no role in study design, data collection and analysis, decision to publish, or preparation of the manuscript.

## Conflict of Interest

The authors declare that the research was conducted in the absence of any commercial or financial relationships that could be construed as a potential conflict of interest.

## Publisher's Note

All claims expressed in this article are solely those of the authors and do not necessarily represent those of their affiliated organizations, or those of the publisher, the editors and the reviewers. Any product that may be evaluated in this article, or claim that may be made by its manufacturer, is not guaranteed or endorsed by the publisher.
